# Source Models of the 2016 and 2022 Menyuan Earthquakes and Their Tectonic Implications Revealed by InSAR

**DOI:** 10.3390/s24113622

**Published:** 2024-06-04

**Authors:** Xixuan Bai, Bingqiang Zhang, Aizhi Guo, Yi Yan, Hao Xu, Xiaoya Bian, Shuwen Zhan, Jiangcheng Chen

**Affiliations:** 1 School of Civil Engineering and Architecture, Wuhan Institute of Technology, 693 Xiongchu Avenue, Wuhan 430074, China; 2 State Key Laboratory of Geodesy and Earth’s Dynamic, Innovation Academy for Precision Measurement Science and Technology, Chinese Academy of Sciences, 340 Xudong Street, Wuhan 430077, China; 3 College of Resources and Environment, South-Central Minzu University, Wuhan 430077, China; 4 College of Earth and Planetary Sciences, University of Chinese Academy of Sciences, 1 Yanqihu East Road, Beijing 100049, China

**Keywords:** Menyuan earthquakes, InSAR, coseismic slip distribution, Haiyuan fault system, tectogenesis

## Abstract

The Haiyuan fault system plays a crucial role in accommodating the eastward expansion of the Tibetan Plateau (TP) and is currently slipping at a rate of several centimeters per year. However, limited seismic activities have been observed using geodetic techniques in this area, impeding the comprehensive investigation into regional tectonics. In this study, the geometric structure and source models of the 2022 Mw 6.7 and the 2016 Mw 5.9 Menyuan earthquakes were investigated using Sentinel-1A SAR images. By implementing an atmospheric error correction method, the signal-to-noise ratio of the 2016 interferometric synthetic aperture radar (InSAR) coseismic deformation field was significantly improved, enabling InSAR observations with higher accuracy. The results showed that the reliability of the source models for those events was improved following the reduction in observation errors. The Coulomb stress resulting from the 2016 event may have promoted the strike-slip movement of the western segment of the Lenglongling fault zone, potentially expediting the occurrence of the 2022 earthquake. The coseismic slip distribution and the spatial distribution of aftershocks of the 2022 event suggested that the seismogenic fault may connect the western segment of the Lenglongling fault (LLLF) and the eastern segment of the Tuolaishan fault (TLSF). Additionally, the western segment of the surface rupture zone of the northern branch may terminate in the secondary branch close to the Sunan-Qilian fault (SN-QL) strike direction, and the earthquake may have triggered deep aftershocks and accelerated stress release within the deep seismogenic fault.

## 1. Introduction

**Figure 1:**
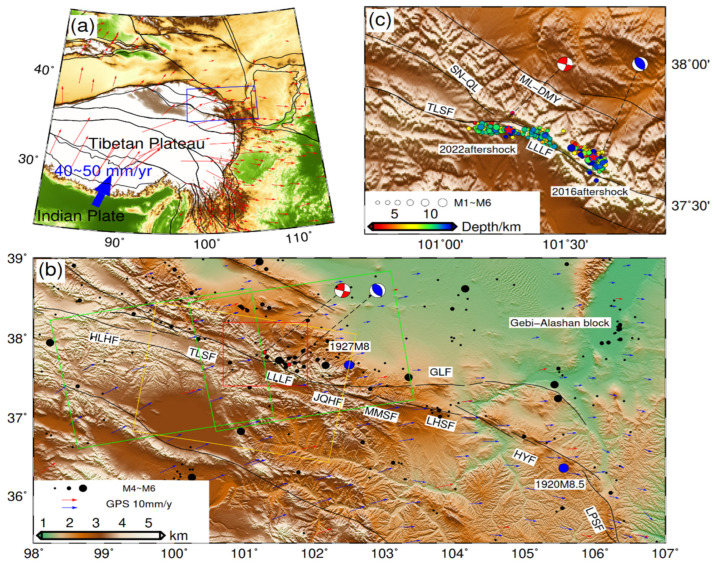
Tectonic settings of the Haiyuan fault system. (**a**) The movement trend of the Tibetan Plateau under the compression of the Indian plate; the blue rectangle is the range of (**b**), and the red arrows are the GPS velocities of the permanent stations [9]. (**b**) The blue and red beach balls represent the focal mechanisms of the 2016 and 2022 events from the United States Geological Survey (USGS), respectively. The black lines represent active faults modified from [9,10], and the solid black dots denote historical earthquakes (1920~2022) from the USGS catalog. Blue arrows indicate the Global Positioning System (GPS) velocities of the roving stations, and red arrows are the GPS velocities of the permanent stations [11]. The green and yellow rectangles indicate the Sentinel-1 ascending and descending track SAR coverages. The red rectangle represents the range of Subfigure (**c**), where the pentagram represents the epicenter of the two main earthquakes after relocation, and the solid point indicates the magnitude and spatial location [12,13].

The continuous collision between the Indian and Eurasian plates has caused the oblique movement of the Tibetan Plateau, leading to the formation of numerous large fault zones [1]. Among these fault zones, the strike-slip Haiyuan fault system, which was formed by the extrusion between the northeastern margin of the TP and the Gebi-Alashan block, played a crucial role in accommodating the eastward expansion of the TP [2,3,4]. Characterized by a sinistral strike-slip movement, the Haiyuan fault system exhibits an overall NW–SE trend, originating from the Halahu fault (HLHF) in the west and extending to the Liupanshan fault (LPSF) in the east (Figure 1) [4]. Within this fault system, a large earthquake of Mw 8.25 occurred in Haiyuan in 1920, and another one of Mw 7.7 occurred in Gulang in 1927 [5,6,7,8]. However, few large earthquakes have been instrumentally recorded in the western segment of the Haiyuan fault system, which limits our understanding of the regional active structures.

Previous geological investigations suggested that the slip rate of the Lenglongling fault (LLLF) was 19 ± 5 mm/yr [14], and the Maomaoshan fault (MMSF) had a slip rate of approximately 12 ± 4 mm/yr [15,16]. This slip rate decreased to 8 ± 2 mm/yr as the fault extended to the easternmost segment of the Haiyuan fault system [3]. Recent studies suggested the strike-slip rate of these fault zones was likely to be overestimated, while a continuous downward trend from west to east persisted [16,17,18]. Multiple segments of the Haiyuan fault system exhibit sinistral strike-slip movements, with a rate of 4.5–9 mm/yr based on GPS observations in recent years [11,19,20]. Nevertheless, InSAR observations during 2016–2021 reveal that the strike-slip rate of the western end of the Haiyuan fault system (close to HLHF) is 1.8 ± 0.3 mm/yr, exhibiting a gradual increase towards the east. This rate reaches 6.4 ± 0.5 mm/yr in proximity to the LPSF and subsequently decreases to 1.3 ± 0.7 mm/yr in the easternmost segment [21]. This relatively low strike-slip rate of the LLLF in recent years suggests that greater strain energy may have accumulated in the area, increasing the seismic risk of the fault zone.

On 21 January 2016, a 6.4 Mw earthquake occurred in Menyuan County, China, with its epicenter located in the northern part of the central Lenglongling Mountains. Approximately six years later, another earthquake with a Mw of 6.9 struck the same area, less than 40 km away from the event, in 2016. Both earthquakes within the Lenglongling area exhibit close spatial and temporal proximity. However, they show different focal mechanisms, reflecting the complexity of the geological structure in this region. Despite extensive seismic observations conducted in this area, accurate recording of earthquakes above M6 by instruments remains rare, and tectonic research is relatively insufficient [5,6,7]. Fortunately, the Sentinel-1A satellite observed the coseismic surface deformations of the two Menyuan earthquakes in recent years. As far as we know, these are the only two seismic events observed by InSAR in the Haiyuan fault system, which provide an opportunity to investigate regional seismic activity and tectogenesis.

Existing studies suggested that the 2016 earthquake was a thrust-slip event [22,23,24]. However, the primary kinematic features of the LLLF exhibit left-lateral movement. Initial field geological surveys revealed that the 2022 Menyuan earthquake led to the formation of two primary rupture zones with a left-step oblique arrangement, spanning approximately 27 km in total length towards the south and north. The maximum strike-slip displacement between these two ruptures reached 3.7 m, and an intervening surface rupture cavity measuring around 3 km was observed [25,26]. The presence of numerous fractures and significant deformation gradients near the fault poses challenges in accurately determining coseismic surface deformations, resulting in different results from existing studies on surface deformation and source models [27,28,29,30,31,32]. It also indirectly hinders investigations into tectonic movements. In addition, the deformations obtained by unwrapping the interference phase are distributed within the range of 50–80 cm [27,28,29], which is significantly smaller than the value discovered in field surveys near fault zones [25,26].

In this study, the coseismic surface deformations of the two earthquakes were derived from Sentinel-1 SAR images. In order to mitigate the influence of measurement errors on slip distribution inversion, atmospheric delays were estimated by fitting the correlation between DEM and noise and subsequently excluded from the 2016 coseismic surface deformation [33,34]. The 2022 earthquake caused the simultaneous movement of multiple nearly parallel sub-faults, resulting in poor coherence of SAR data in some areas adjacent to fractures and posing challenges for the retrieval of the wrapped phase [25,26,31]. Therefore, we processed the SAR data covering the 2022 coseismic deformation area and masked some of the incoherent phases near the fault. The measured data before and after masking the low coherence phase were used for the inversion of coseismic slip distribution, which was then combined with the early field geological survey [25,26] and the spatial distribution of aftershocks [20,21], with the aim to investigate the source models of both events and elucidate tectonic movement characteristics of seismogenic faults.

## 2. Data and Methods

We collected five pairs of Sentinel-1A images from the European Space Agency for this study, and the corresponding data information is presented in Table 1. The images of the ascending track (T128) and descending track (T33) fully covered the coseismic range of the two events. Although the image from another ascending track (T26) covered only half the range of the 2022 event, it had a significantly higher radar incidence angle in the center of the deformation field (about 45.3 degrees) than the images of the T128 (about 36.6 degrees), which still provided effective constraint (the image coverage range is shown in Figure 1).

### 2.1. Coseismic Deformations and InSAR Measurement Errors Correction of Menyuan 2016 Earthquake

**Figure 2:**
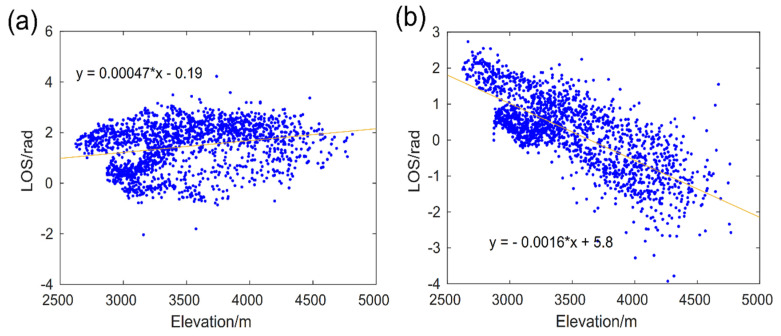
The function models of atmospheric delay correction. The blue scatter represents the distribution of noise and elevation, and the yellow solid line represents the function based on the fit between noise and elevation; (**a**) is the function model of the ascending track (T128), (**b**) is the function model of the descending track (T33).

The GMTSAR [35,36] was employed to process the SAR data covering the coseismic range of the 2016 event, and the SNAPHU algorithm was used to unwrap the interferogram [37]. The results showed that both ascending and descending tracks exhibited uplift in the line of sight (LOS), indicating that no surface rupture occurred during the coseismic deformations. In addition to the coseismic deformation, significant noise levels were also observed in the study area. Given that the coseismic region is located within the Lenglongling Mountains, with significant topographic relief, we speculate that these noises may be signal delays caused by atmospheric water vapor related to the topography. Furthermore, owing to the relatively small deformations, these noises could potentially impact the final results of the inversion for coseismic slip distribution. As a result, a function model is fitted between noise and elevation in the surrounding area with almost no coseismic deformations (as shown in Figure S1 and Figure 2). Subsequently, atmospheric delays were corrected for the entire deformation range based on this model. It is worth noting that we excluded the area near the epicenter during the function fitting process in order to avoid the effect of real coseismic deformation signals on the function model between noise and elevation. After subtracting the atmospheric delay signal and orbit-related errors, the noise of the InSAR coseismic surface deformation field is significantly reduced, especially with respect to the significant distribution of atmospheric effects in the descending track of the deformation field (Figure S1). Additionally, statistical parameters such as root mean square and standard deviation indicate a significant reduction in far-field noise after correcting measurement errors across the coseismic deformation field (Table S1).

### 2.2. Coseismic Surface Deformations of the Menyuan 2022 Earthquake

**Figure 3:**
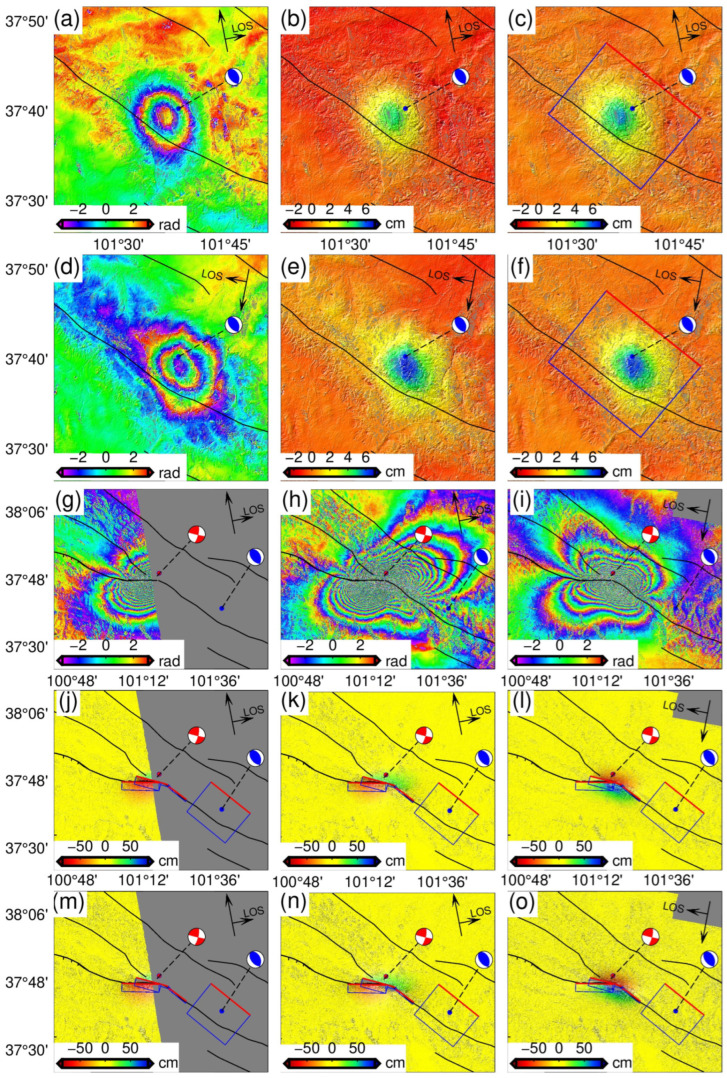
Coseismic surface deformations of the Menyuan earthquakes in 2016 and 2022. (**a**,**d**) Coseismic interferograms of the 2016 earthquake, (**b**,**e**) the line-of-sight (LOS) displacements for radars obtained from these interferograms, (**c**,**f**) line-of-sight (LOS) displacements after correcting atmospheric delays. (**g**–**i**) The coseismic interferogram of the 2022 earthquake, (**j**–**l**) the line-of-sight (LOS) displacements obtained from these interferograms, and (**m**–**o**) the deformations after masking the area of low coherence. The black solid line represents the fault trace of the modified reference [17,18], the red beach ball represents the focal mechanism of the 2022 event, and the blue ball represents the 2016 event. The rectangle formed by the blue and red lines is the vertical projection of the fault plane on the surface, where the red line represents the top boundary of the fault plane.

Three pairs of SAR images (from T26, T33, and T128) were selected to obtain the coseismic surface deformation of the 2022 Menyuan earthquake. The radar signal coherence in the epicenter rupture area was exceptionally weak, as evidenced by the interferogram (as shown in Figure 3g–i) and the radar image coherence (Figure S2). Initially, we employed the SNAPHU algorithm [36] for phase unwrapping, which is a global unwrapping algorithm. The resulting deformation field exhibits continuous gradients between any two positions along any given path. However, according to the results of the field geological survey, the earthquake caused a significant rupture on the surface with a left-lateral strike slip up to 3.7 m [25], indicating that the data distributed on the hanging wall and foot wall of the fault may have a maximum phase jump equivalent to a 3.7 m relative dislocation when crossing the fault. We employed GMTSAR and ISCE [38] to perform interferogram unwrapping based on the SNAPHU algorithm. In order to satisfy the restriction for continuous gradients in all directions, the obtained dislocations along the strike of the fault were minimized to approach zero [39]. Additionally, the maximum LOS displacements were about 65 cm (Figure S3). The InSAR measurements are significantly inconsistent with the surface deformation observed during the field geological survey. This discrepancy may be attributed to the decrease in the deformation gradient across the entire deformation field, as the values near the fault tend towards zero. Therefore, it can be concluded that for an event with significant surface rupture and low coherence near the rupture zone, an unwrapped interferogram based on the traditional SNAPHU algorithm leads to small deformation values [40,41].

## 3. Results

PSOKINV5.0 software [42,43,44,45] was employed for geodetic modeling. Due to the high sampling frequency and large number of pixels in SAR images, it was necessary to ensure both feasibility and efficiency of the calculation process. Hence, the Quadtree algorithm [46] was adopted to downsample the InSAR deformation signal. The downsampled deformation field before and after masking the low coherence phase is shown in Figure S4.

### 3.1. Coseismic Slip of 2016 Event

**Figure 4:**
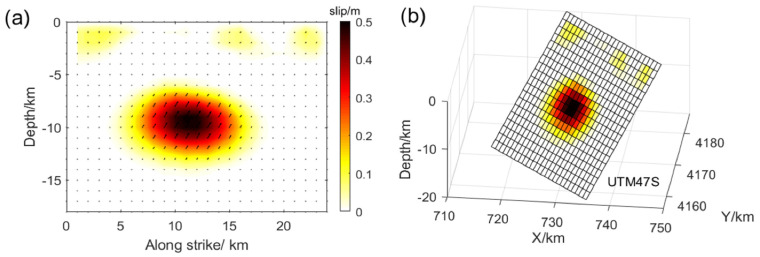

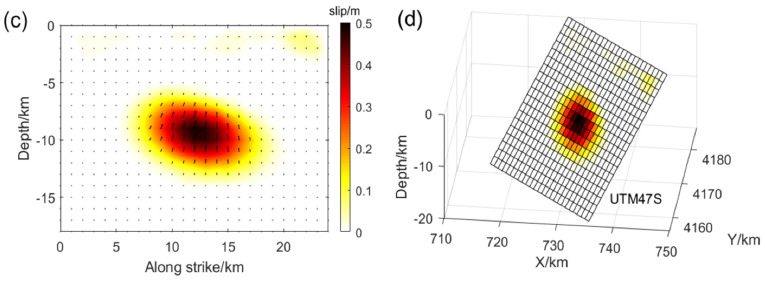
Coseismic slip distribution corresponding to events in 2016; the square represents a discrete sub-fault, and the black arrow represents the slip vector; (**a**,**b**) show the coseismic slip distribution before atmospheric delay correction; (**c**,**d**) display the coseismic slip distribution after atmospheric delay correction.

The SAR image processing of T128 and T33 indicates that the coseismic surface deformation observed during the 2016 earthquake was characterized by uplift, suggesting a potential thrust-slip event occurring on a blind fault. To investigate the coseismic slip distribution under the constraint of InSAR observations, a two-step inversion strategy was adopted. First, the rectangular fault with uniform slip was determined by minimizing the square misfit between the simulated and observed coseismic deformations based on particle swarm optimization (PSO) and elastic dislocation theory [47]. Afterward, the fault was extended and discretized into multiple sub-faults of 1 km × 1 km, followed by an inversion to determine the distribution of coseismic slip. The inversion was initially performed with the deformation field without error correction as the constraint, resulting in fault dislocation exhibiting dip slip along almost a single rake angle (Figure 4a,b). At the same time, several small slips were scattered near the fault boundary in addition to the main slip area, exhibiting a distribution similar to previous studies [30]. Therefore, we corrected the InSAR deformation field for atmospheric delays and fitted orbital-dependent errors. Finally, the source parameter inversion was performed using constraints from the error-corrected InSAR observations.

The error-corrected coseismic deformation field was used in the inversion to attenuate the influence of InSAR measurement errors. It can be observed that several discrete slips were weakened (Figure 4), and the atmospheric noise in the coseismic surface deformation was reduced. The slip patches of the source model were mainly distributed in the depth range of 7~13 km, with a maximum slip of about 0.5 m. A trend from dip-slip to thrust can be observed from the east and west edges to the center of fault planes (Figure 4c,d), corresponding to the recent crustal shortening and uplift of the LLLF zone revealed by InSAR [16]. In addition, we assume that with the shear modulus of 30 GPa, the estimated seismic moment was M_0_ = 1.131 × 10^18^ N∙m and corresponded to Mw 5.9, consistent with the results from Global Centroid Moment Tensor Project and the U.S. Geological Survey. The coseismic slip distribution indicates that the presence of atmospheric noise can introduce significant errors in coseismic inversion for seismic events with small InSAR LOS deformation, such as the 2016 Menyuan event with a maximum deformation value of less than 7 cm. The results of the 2016 event demonstrate that correcting for atmospheric delays in InSAR observation data can mitigate the error and yield a more accurate representation of the fault movement characteristics through improved coseismic slip distribution. Therefore, it is crucial to consider atmospheric interference in seismic studies, especially those involving small-scale surface deformations.

### 3.2. Fault Geometry of 2022 Event

**Figure 5:**
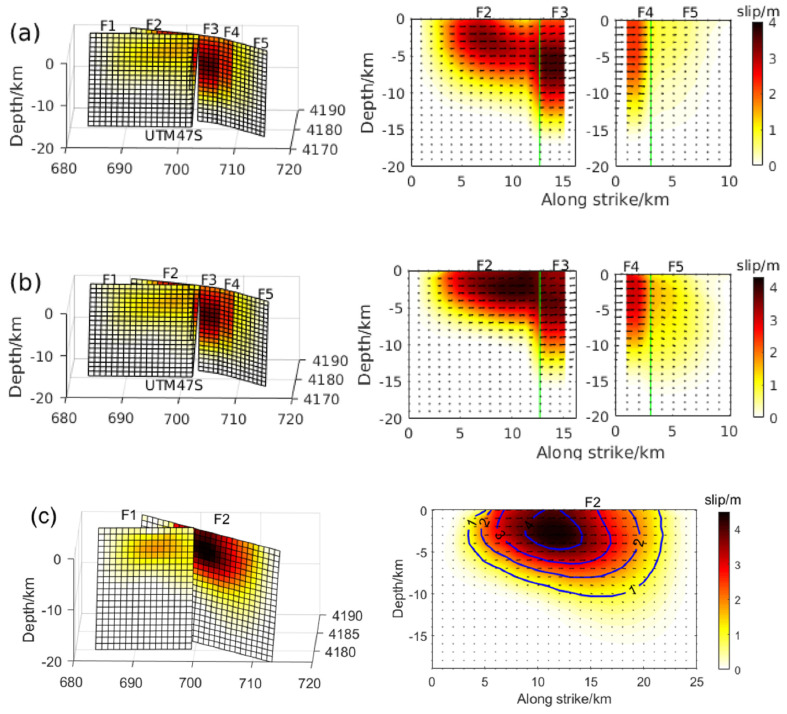
Coseismic slip distribution corresponding to events in 2022; the square represents a discrete sub-fault, and the black arrow represents a slip vector; (**a**) is the coseismic slip distribution model 1, determined by the initial InSAR observations; (**b**) is the coseismic slip distribution model 2, obtained by the coseismic deformations after masking the low-coherence area; (**c**) is model 3 and replaces the F2–F5 segments in (**b**) with the F2 segment.

Field geological surveys [25,26] have revealed that two major fault zones in the north and south of the surface induced by the earthquake consisted of tensile and shear fractures. The maximum strike-slip displacement of the southern branch rupture was about 85 cm, with a strike angle ranging from 91° to 93°. Because of the relatively small dislocations and the small number of joint motion fractures, the SAR image did not result in significant low coherence, and the fracture trace of the fault can be identified in the interferogram (F1, as shown in Figure 3). The maximum strike-slip displacement of the north branch fault was 3.7 m, with its strike direction gradually changing from 102° to 120° from west to east [25]. The combined movement of various structural fractures distributed along the main rupture zone, combined with significant deformation gradients, was possibly responsible for the low coherence observed in the near-fault areas of the interferogram, hindering accurate identification of fault traces and recovery of near-fault coseismic deformations from the wrapped phase. However, errors caused by phase unwrapping in the low-coherence areas can be avoided through POT, which determined the change in strike direction of these faults to be from 103.5° to 127° from west to east [31]. In addition, both InSAR observations and field investigation [25,26] suggest a rapid change in the strike direction of the surface trace of the north branch fault between F3 and F4 (Figure 3). This variation in strike direction is further supported by the spatial position obtained by the relocated aftershock [20] (Figure 1c). On this basis, the primary segment of the northern branch rupture zone was divided into two faults: a 12.7 km fault (F2) and an 8 km fault (F5). Additionally, two small faults of 3.5 km (F3) and 3.1 km (F4) in the central part of the fracture zone were connected and transitioned to minimize the discontinuity of the coseismic slip model caused by the rapid change in strike direction in inversion. The surface projection is shown in Figure 3, and the spatial distribution is shown in Figure 5.

To obtain more reliable fault dip angles for the source model, the constraints on the fault dip were imposed by reference to the spatial distribution of aftershocks during InSAR inversion. Considering the nearly coincident surface rupture in the eastern segment of the north branch fault with the surface projection of the relocated aftershock, the dip angles of F5 and F4 were determined to be 88°, and the maximum rupture depth was extended to 20 km to ensure the inclusion of all the slip area during the slip distribution inversion. When the rupture zone extended to the intersection of the LLLF and the SN-QL on the west side, the strike direction revealed by the aftershock distribution changed significantly, and its vertical projection on the surface did not coincide with the location of maximum surface dislocation distribution (Figure 1c and Figure 2j–o). The analysis of the surface rupture trace and the spatial distribution of aftershocks reveals significant changes in both surface strike direction and dip angle as the coseismic rupture propagated westward along the LLLF. The dip angle for fault F1 was set to 78° based on the distribution of aftershocks to ensure that the projection of the fault plane on the surface covers the area of aftershocks. According to the InSAR observations, the fracture zone F2 in the western Lenglongling segment appears to extend towards the SN-QL. However, no aftershocks were observed in this direction. Therefore, we employed the ABIC algorithm [48] to search for the optimal dip angle of F2 within the range of 75–88° with a step size of 1° after determining the angles for other faults (Figure S5).

### 3.3. Coseismic Slip Distribution Model of 2022 Event

To investigate the influence of the low-coherence areas near the fault on the slip distribution model, the initial InSAR and masked low-coherence observations were used as constraints for coseismic slip distribution inversion. The obtained slip distribution model 1 is shown in Figure 5a, and model 2 is illustrated in Figure 5b. According to the spatial distribution of aftershocks and the coseismic observations, the constant change in the strike and dip angle of the seismogenic fault can be observed. Although dividing the fault into multiple sub-faults enhances the fidelity of the source model to the actual seismogenic fault structure, it also compromises the spatial continuity of slip distribution on the fault plane. Hence, we introduced a continuous fault plane (model 3, as shown in Figure 5c) to validate the previous slip distribution and examine the reliability of the multi-fault slip distribution model. In this model, F1 is kept constant, the strike of F2 is determined to be 106° based on the fracture track, and the dip angle is finally determined to be 84° after searching with the ABIC algorithm in 1° steps within the range of 78° to 88°, which is similar to that of the double-fault model in the previous study [32].

**Figure 6:**
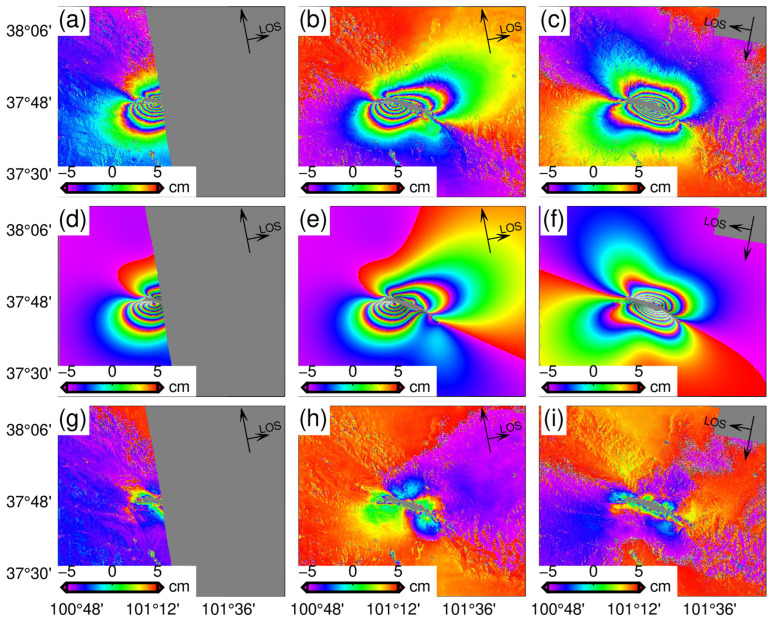
Coseismic deformation and residual based on coseismic slip distribution forward modeling of the 2022 Menyuan earthquake; (**a**–**c**) illustrate the InSAR observations, (**d**–**f**) show the simulation results, and (**g**–**i**) display the residuals.

The inversion of the coseismic slip distribution shows that the maximum slip for model 1 is approximately 3.8 m. The center of the slip is located at a depth of 4~6 km, and the slip area gradually expands to the depth (Figure 4) as it extends from west to east along the LLLF. The maximum surface dislocation revealed by the slip distribution model is less than 2 m, which is significantly smaller than the maximum slip of the near-fault area during the field geological investigation [25,26]. This difference could be attributed to incomplete recovery of the wrapped phase in the areas of low coherence near the fault. The slip trends of model 2 and model 1 are similar, with the western segment (F2) showing a slip range of about 0~9 km and the eastern segments (F3~F5) demonstrating a slip range of about 0~16 km. This slip distribution trend suggests that the rupture of the seismogenic fault varies from shallow to deep from west to east. Meanwhile, both model 2 and model 3 exhibit a maximum slip depth of 2~3 km after masking the low-coherence areas near the fault based on InSAR observations. There is a rupture belt about 6 km long with a relative dislocation of 3 m, which is similar to the results of field geological investigation [24]. The results of the forward simulation based on the slip distribution of model 2 are in good agreement with InSAR observations (Figure 6). In addition, F2 was used instead of F2–F5 to invert the continuous fault plane in model 3 (Figure 5c). Notably, the coseismic slip distribution trend is similar to the previous two models, indicating that the multi-fault model remains reliable despite discontinuity points when considering aftershock distribution and surface rupture traces.

### 3.4. Coulomb Stress Changes Caused by Earthquakes in 2016 and 2022

**Figure 7:**
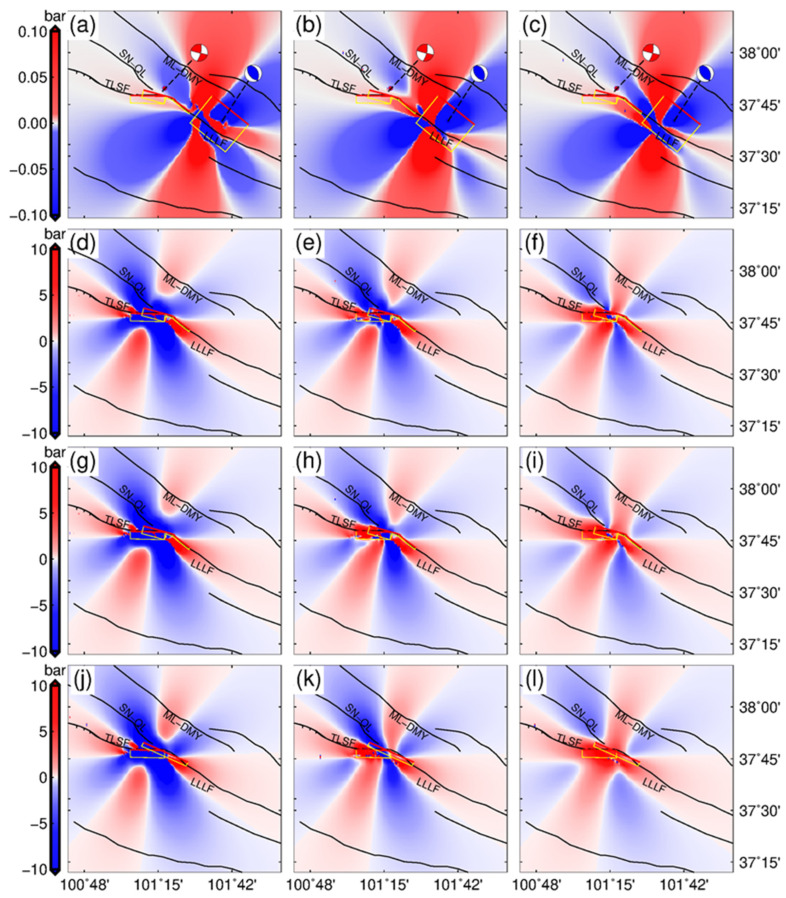
Coulomb stress changes caused by the two earthquakes; (**a**–**c**) Coulomb stress changes at 5 km, 10 km, and 15 km caused by the 2016 earthquake; (**d**–**f**) Coulomb stress changes caused by the coseismic slip distribution in Figure 5a; (**g**–**i**) those corresponding to coseismic slip distribution in Figure 5b; (**j**–**l**) those corresponding to Figure 5c. The depth from left to right is 5 km, 10 km, and 15 km. The solid black lines represent the fault traces modified based on the literature [9,10] (Deng et al., 2003; Zhang et al., 2002).

In order to investigate the possible triggering mechanism of the 2016 earthquake on the 2022 earthquake and assess the induced changes in regional tectonic stress changes (∆CFS) due to coseismic slip during the 2022 event, the Coulomb stress change caused by the 2016 and 2022 Menyuan earthquakes is calculated using Equation (1), with positive ∆CFS indicating that it promotes the tendency of fault rupture [49,50]:

∆CFS = ∆*τ* + *μ*∆*σ_n_* (1)

where ∆*σ_n_* represents the change in normal stress on the fault, which is positive along the direction of tension; ∆*τ* denotes the change in shear stress on the fault, which is the fault slip direction; *μ* is the effective friction coefficient. Firstly, we calculated the Coulomb stress changes caused by the 2016 earthquake using the seismogenic fault of the 2022 earthquake as the receiving fault. The calculation area was expanded to ensure that the range of stress disturbance caused by the 2016 event was included. To reflect the Coulomb stress changes in the underground space region caused by coseismic slip, we calculated the Coulomb stress changes at different depths parallel to the surface. The calculation results are shown in Figure 7.

## 4. Discussion

### 4.1. Source Model of the 2016 Event and Its Impact on the 2022 Event

InSAR observations are susceptible to various sources of error, including atmospheric propagation delay, aftershocks, and ionospheric disturbances [33,34]. We made atmospheric corrections by leveraging the correlation between near-surface atmospheric water vapor and topography, fitting this correlation with the DEM. Errors from the ionosphere and aftershocks, which are unrelated to the DEM, were found to be negligible in our study. According to the coseismic slip distribution of the 2016 event obtained under the constraint of InSAR coseismic surface deformation with atmospheric delays, the fault dislocation follows almost a single rake angle, and significant slip exists outside the fault plane center region. Since such discrete and discontinuous slip distribution is usually found in large fault planes and is triggered by strong earthquakes, this distribution is unreasonable for a 6.4 Mw earthquake occurring on blind faults. Therefore, a reliable coseismic deformation is obtained by measurement error correction (Figure 3c,f), and the coseismic slip distribution inversion constrained by the deformation field shows that the east and west slip edges extrude towards the epicenter, forming thrust slip eventually. The coseismic slip is primarily in the 6 to 14 km depth range, the slip angle along the strike of the fault gradually changes from 70° to 110°, and the slip center is a pure thrust slip with a rake angle of about 90°. The slip distribution trend is more consistent with the characteristics of local crustal shortening and the LLLF zone uplift revealed by recent geodetic investigations [16,30]. Thus, atmospheric noise may hinder the establishment of the optimal focal mechanism and coseismic slip distribution model through InSAR inversion for seismic events with centimeter-level deformations, the effect of which should be considered when using InSAR deformation to study earthquakes with small deformations.

Although the two Menyuan earthquakes showed very close spatiotemporal distributions, they exhibited different coseismic activity patterns and mechanisms, reflecting the complexity of regional geological structures. Coulomb stress changes were calculated using the 2016 coseismic slip distribution model as the source fault and the 2022 seismogenic fault as the receiving fault to investigate the impact of the 2016 earthquake on the latter [49,50]. The Coulomb stress changes were calculated at depths of 5 km, 10 km, and 15 km, respectively (Figure 7), with the results indicating the greatest changes occurring at a depth of 10 km. In addition, the 2016 earthquake caused an increased Coulomb stress in the western and eastern segments of the LLLF, and the seismogenic fault of the 2022 earthquake was almost completely in the area with Coulomb stress increments caused by the 2016 earthquake within the 5–15 km depth. Hence, the 2016 earthquake promoted the occurrence of the 2022 one.

### 4.2. Regional Tectogenesis Revealed by the 2022 Menyuan Earthquake

Some past controversy was raised about the trace of the western LLLF. The Institute of Geology and Lanzhou Institute of Seismology believed that the LLLF extended westward and connected with the SN-QL [51,52], while Taylor and Xu et al. believed that the strike direction of the western Lenglongling segment changed significantly and connected with the TLSF [52,53]. According to the coseismic surface deformations revealed by field geological survey and SAR images, the surface rupture strike of the western segment of LLLF extends along the SN-QL, and rupture branches with smaller dislocation are observed along the west LLLF segment to the TLSF. The coseismic slip distribution model of the 2022 event reveals that the seismogenic fault has a large slip along the strike direction of the SN-QL, but the slip distribution is relatively shallow, and the main slip distribution is concentrated within the 9 km depth range, markedly different from the eastern segments (about 16 km; F4–F5). Moreover, the F1 segment extending along the western side of the LLLF to the east of the TLSF has a small relative dislocation on the surface, but the slip distribution of the F1 segment has a deep slip similar to the F4 and F5 branches on the F1 fault, with a maximum slip of about 1.5 m. The spatial location of the relocated aftershock (Figure 1c) changes significantly along the westward extension of the seismogenic fault and spreads along the strike direction close to the TLSF. Considering these analyses, the coseismic slip model and aftershock spatial distribution of the event suggest that the western section of the LLLF may be connected to the TLSF.

In addition, the coseismic slip distribution model shows no deep slip along the SN-QL direction in the western segment of Lenglongling, i.e., the LLLF may not be connected with the SN-QL in the western section. Previous studies suggested that the main surface rupture zone generated by an earthquake could terminate at the fault branch [54]. Based on the above analyses, the seismogenic fault of the 2022 Menyuan earthquake connects the west Lenglongling segment and the east TLS segment, and the western segment of the surface rupture zone of the northern branch may terminate on the secondary branch with less strike direction change along the SN-QL. The possible reason is that the strike direction of the seismogenic fault varies greatly. Moreover, recent geodetic investigations also showed a continuous left-lateral strike direction slip between the TLSF and LLLF.

### 4.3. Tectonic Implications of the 2016 and 2022 Menyuan Earthquakes

While global teleseismic records and GNSS inversion can swiftly determine source parameters for major events, these methods have limitations in resolving detailed fault slip distributions. Our study leverages InSAR observations to achieve high-precision, high-resolution deformation measurements near the fault. Unlike the sparse distribution of GNSS observations, InSAR allows for accurate inversion of coseismic slip distributions on seismogenic faults. This precision is crucial for subsequent stress field calculations and tectonic movement studies. Based on the inversion of the InSAR deformation field after observation error correction, the coseismic slip distribution of the 2016 event is more consistent with the crustal movement trend. Therefore, the slip distribution in Figure 4c,d is more reasonable. Analysis of the coseismic slip distribution of the 2016 earthquake indicates that the earthquake mainly released the elastic energy accumulated by vertical strain during the crustal shortening and uplift movement of the LLLF. The thrust slip deep in the fault may have intensified the stress concentration in the shallow, unruptured area. The resulting stress changes promoted the strike-slip movement of the LLLF zone and the occurrence of the 2022 earthquake. In addition, recent InSAR time series observations indicate a continuous slip between the LLLF and TLSF [16], a trend also reflected in the coseismic slip distribution model constrained by InSAR surface deformation observations of the 2022 event in this study. Therefore, the LLLF may be connected to the TLSF, and the slip distribution indicates that the 2022 earthquake mainly released the elastic energy accumulated in the shallow part of the LLLF zone.

Our conclusions are further supported by long-term GNSS observations and precise aftershock localization from global teleseismic records. The rise of deep Coulomb stress caused by shallow coseismic rupture could trigger deep aftershocks, release accumulated stress, and reduce the risk of future strong earthquakes. Alternatively, the loading of deep faults due to these stress changes could cause further stress concentration, increasing the risk of future strong earthquakes [31,55]. The aftershocks of the 2022 event concentrated primarily at 10 km (Figure 1c), with some aftershocks reaching the depth of the 2016 event. However, the coseismic slip center was about 3 km to 5 km deep in the shallow part of the fault. Therefore, the event may have triggered aftershocks in deeper regions, releasing the elastic strain energy accumulated during the deep part convergence of the LLLF and reducing the risk of future strong earthquakes. Previous studies suggested that the recent interseismic strike-slip rate of the LLLF zone has been about 4–6 mm/yr, with a shortening rate of about 1 mm/yr [16]. Additionally, the coseismic slip pattern and strain partitioning may be influenced by the ratio of interseismic strike-slip and shortening rates and regional fault geometry [30]. It is inferred that the elastic strain energy of the fault is released with a left-lateral strike slip and a small amount of thrust slip, according to the coseismic slip distribution of the 2022 event, increasing the Coulomb stress of the Jinqianghe fault in the east and the TLSF in the west (Figure 1 and Figure 7), and it may have released the stress in the deep part of the LLLF. Meanwhile, the 2022 Menyuan earthquake also increased the Coulomb stress on the east and west sides of the LLLF zone (Figure 7), possibly promoting the tectonic movement of the western Haiyuan fault system. In addition, early studies suggested that the slip rates of the Haiyuan fault system had a downward trend from west to east [4,11,12,14]. However, the InSAR time series surface observations revealed that the recent-year slip rates of the TLSF were below that of the eastern segments of the Haiyuan fault system [16]. Hence, a large amount of elastic strain energy may have been accumulated in this region. Moreover, increased Coulomb stress in the region due to the 2022 event may further increase earthquake risks.

## 5. Conclusions

To obtain the coseismic surface deformations of the 2016 Menyuan earthquake, this study applied an atmospheric error correction method for coseismic surface deformations aiming at seismic events with small deformations. Subsequently, the more reliable source models of the 2016 and 2022 Menyuan earthquakes were obtained through inverting InSAR coseismic observations, and the tectonic movement characteristics of the western segment of the Haiyuan fault system were investigated. The main findings include the following:

(1) Using the atmospheric error-corrected InSAR deformation field as a constraint, more reliable source mechanisms and coseismic slip distributions were obtained for the 2016 event, indicating that atmospheric errors may significantly degrade the signal-to-noise ratio of the coseismic deformation field for events with small deformation magnitudes, thus validating the effectiveness of the proposed approach.
(2) The 2016 event may have released the elastic strain energy accumulated during the crustal shortening of the LLLF zone while promoting the left-lateral strike-slip movement of the western segment of the LLLF zone and the occurrence of the 2022 Menyuan earthquake.
(3) The seismogenic fault of the 2022 event is the western segment of the LLLF and the eastern section of the TLSF. Additionally, the western segment of the surface rupture zone from the northern branch may terminate in the secondary branch close to the strike direction of the SN-QL.
(4) The focal mechanisms of the two Menyuan earthquakes completely differed, which may have respectively released the elastic strain energy accumulated by the shallow strike slip and the deep crust shortening of the LLLF zone caused by the eastward expansion of the TP. The 2022 event also reduced the stress in the deep part of the LLLF zone, and future attention should be paid to earthquakes in the TLSF and JQHF.

## Figures and Tables

**Table 1:** Basic parameters of SAR image.

Event	Orbit Direction	Track	Master–Slave Date	Incident Angle (°)	Azimuth Angle (°)
2016	Ascending	T128	2016/01/13–2016/02/06	37.6	−10
	Descending	T33	2016/01/18–2016/02/11	36.1	−169
2022	Ascending	T128	2022/01/05–2022/01/17	36.6	−169
	Ascending	T26	2021/12/29–2022/01/10	45.3	−169
	Descending	T33	2021/12/29–2022/01/10	38	−10

## Data Availability

Sentinel-1 SAR images were downloaded from ESA (https://scihub.copernicus.eu/dhus/#/home (accessed on 20 January 2022)). The source model inversion was completed using PSOKINV software (https://github.com/wpfeng/psokinv/tree/master (accessed on 10 January 2022)). The coseismic slip models of the 2016 and 2022 Menyuan earthquakes are stored in GitHub and can be accessed online (https://github.com/zhang785/menyuan-earthquake (accessed on 14 September 2023)). All figures were plotted using Generic Mapping Tools version 5.4 (https://www.generic-mapping-tools.org (accessed on 10 September 2020)). Other datasets and software cited in this article are listed in the References section.

## Supplementary Materials

The following supporting information can be downloaded at https://www.mdpi.com/article/10.3390/s24113622/s1, Supplementary Material Figure S1: The function model and result of atmospheric error correction, Supplementary Material Figure S2: The coherence of radar signals requires that the coherence of the red inside of the mask is much lower than that of the surrounding area, Supplementary Material Figure S3: Results of processing using the SNAPHU algorithm, Supplementary Material Figure S4: Coseismic deformations after downsampling, Supplementary Material Figure S5: The optimal dip angle of F2 in a multi-fault model for the 2022 earthquake, Supplementary Material Table S1: Statistical parameters of 2016 Menyuan earthquake coseismic deformations error before and after correction.

## References

[1] Tapponnier P., Zhiqin X., Roger F., Meyer B., Arnaud N., Wittlinger G., Jingsui Y. (2001). Oblique stepwise rise and growth of the Tibet Plateau. Science.

[2] Burchfiel B.C., Zhang P., Wang Y., Zhang W., Song F., Deng Q., Molnar P., Royden L. (1991). Geology of the Haiyuan fault zone, Ningxia-Hui Autonomous region, China, and its relation to the evolution of the northeastern margin of the Tibetan Plateau. Tectonics.

[3] Zhang P., Peter M., Burchfiel B.C., Royden L., Wang Y., Deng Q., Song F., Zhang W., Jiao D. (1998). Bounds on the Holocene slip rate of the Haiyuan fault, north-central China. Quat. Res..

[4] Zhu L., Wang C., Zheng H., Xiang F., Yi H., Liu D. (2006). Tectonic and sedimentary evolution of basins in the northeast of Qinghai-Tibet Plateau and their implication for the northward growth of the Plateau. Palaeogeogr. Palaeoclimatol. Palaeoecol..

[5] Ou Q., Kulikova G., Yu J., Elliott A., Parsons B., Walker R. (2020). Magnitude of the 1920 Haiyuan earthquake reestimated using seismological and geomorphological methods. J. Geophys. Res. Solid Earth.

[6] Guo P., Han Z.J., Jiang W.L., Mao Z.B. (2017). Holocene Left-Lateral Slip Rate of the Lenglongling Fault, Northeastern Margin of the Tibetan Plateau. Seismol. Geol..

[7] Gu G. (1989). Catalogue of Chinese Earthquakes.

[8] Li K., Tapponnier P., Xu X., Kang W. (2023). The 2022, Ms 6.9 Menyuan earthquake: Surface rupture, Paleozoic suture re-activation, slip-rate and seismic gap along the Haiyuan fault system, NE Tibet. Earth Planet. Sci. Lett..

[9] Zhang P., Wang Q., Ma Z. (2002). GPS Velocity Field and Active Crustal Blocks of Contenporary Tectonic Deformation In Continental China. Earth Sci. Front..

[10] Deng Q., Zhang P., Ran Y., Min W., Yang X., Chu Q. (2003). Basic characteristics of active tectonics of China. Sci. China.

[11] Zhao B., Huang Y., Zhang C., Wang W., Tan K., Du R. (2015). Crustal deformation on the Chinese mainland during 1998–2014 based on GPS data. Geod. Geodyn..

[12] Fan L., Li B., Liao S., Jiang C., Fang L. (2022). Precise relocation of the aftershock sequences of the 2022 M6.9 Menyuan earthquake. Earthq. Sci..

[13] Liang S., Lei J., Xu Z., Zou L., Liu J. (2017). Relocation of the aftershock sequence and focal mechanism solutions of the 21 January 2016 Menyuan, Qinghai, M S 6.4 earthquake. Chin. J. Geophys..

[14] Lasserre C., Gaudemer Y., Tapponnier P., Mériaux A.-S., Van der Woerd J., Daoyang Y., Ryerson F.J., Finkel R.C., Caffee M.W. (2002). Fast late pleistocene slip rate on the leng long ling segment of the Haiyuan fault, Qinghai, China. J. Geophys. Res..

[15] Lasserre C., Morel P.-H., Gaudemer Y., Tapponnier P., Ryerson F.J., King G.C.P., Métivier F., Kasser M., Kashgarian M., Liu B. (1999). Postglacial left slip rate and past occurrence of M ≥ 8 earthquakes on the western Haiyuan fault, Gansu, China. J. Geophys. Res..

[16] Li C., Zhang P.Z., Yin J., Min W. (2009). Late Quaternary left-lateral slip rate of the Haiyuan fault, northeastern margin of the Tibetan Plateau. Tectonics.

[17] Jolivet R., Lasserre C., Doin M., Guillaso S., Peltzer G., Dailu R., Sun J., Shen Z., Xu X. (2012). Shallow creep on the Haiyuan fault (Gansu, China) revealed by SAR Interferometry. J. Geophys. Res..

[18] Shao Y., Liu-Zeng J., Van der Woerd J., Klinger Y., Oskin M.E., Zhang J., Wang P., Wang P., Wang W., Yao W. (2021). Late Pleistocene slip rate of the central Haiyuan fault constrained from optically stimulated luminescence, 14C, and cosmogenic isotope dating and high-resolution topography. GSA Bull..

[19] Gan W., Zhang P., Shen Z., Niu Z., Wang M., Wan Y., Zhou D., Cheng J. (2007). Present-day crustal motion within the Tibetan Plateau inferred from GPS measurements. J. Geophys. Res..

[20] Wang M., Shen Z.K. (2020). Present-day crustal deformation of continental China derived from GPS and its tectonic implications. J. Geophys. Res. Solid Earth.

[21] Huang Z., Zhou Y., Qiao X., Zhang P., Cheng X. (2022). Kinematics of the ~1000 km Haiyuan fault system in northeastern Tibet from high-resolution Sentinel-1 InSAR velocities: Fault architecture, slip rates, and partitioning. Earth Planet. Sci. Lett..

[22] Zhang Y., Shan X., Zhang G., Zhong M., Zhao Y., Wen S., Qu C., Zhao D. (2020). The 2016 Mw 5.9 Menyuan Earthquake in the Qilian Orogen, China: A Potentially Delayed Depth-Segmented Rupture Following from the 1986 Mw 6.0 Menyuan Earthquake. Seismol. Res. Lett..

[23] Wang H., Liu-Zeng J., Ng A.-M., Ge L., Javed F., Long F., Aoudia A., Feng J., Shao Z. (2016). Sentinel-1 Observations of the 2016 Menyuan Earthquake: A Buried Reverse Event Linked to the Left-Lateral Haiyuan Fault. Int. J. Appl. Earth Obs. Geoinf..

[24] Li Y., Jiang W., Zhang J., Luo Y. (2016). Space geodetic observations and modeling of 2016 Mw 5.9 Menyuan earthquake: Implications on seismogenic tectonic motion. Remote Sens..

[25] Pan J., Li H., Chevalier M.-L., Liu D., Li C., Liu F., Wu Q., Lu H., Jiao L. (2022). Surface rupture zone and seismogenic structure of the 2022 Menyuan Ms6.9 earthquake in Qinghai Province. Acta Geol. Sin..

[26] Han S., Wu Z.H., Gao Y., Lu H.F. (2022). Surface rupture investigation of the 2022 Menyuan MS 6.9 earthquake, Qinghai, China: Implications for the fault behavior of the Lenglongling fault and regional intense earthquake risk. J. Geomech..

[27] Li Z., Han B., Liu Z., Zhang M., Yu C., Chen B., Liu H., Du J., Zhang S., Zhu W. (2022). Source parameters and slip distributions of the 2016 and 2022 Menyuan, Qinghai earthquakes constrained by InSAR observations. Geomat. Inf. Sci. Wuhan Univ..

[28] Yang H., Wang D., Guo R., Xie M., Zang Y., Wang Y., Yao Q., Cheng C., An Y., Zhang Y. (2022). Rapid report of the 8 January 2022 Ms 6.9 Menyuan earthquake, Qinghai, China. Earthq. Res. Adv..

[29] Bao X., Zhang R., Wang T., Shama A., Zhan R., Lv J., Wu R., Fu Y., Liu G. (2022). The Source Mechanism and Fault Movement Characterization of the 2022 Mw6.7 Menyuan Earthquake Revealed by the Joint Inversion with InSAR and Teleseismic Observations. Front. Environ. Sci..

[30] Luo H., Wang T. (2022). Strain partitioning on the western Haiyuan fault system revealed by the adjacent 2016 Mw5.9 and 2022 Mw6.7 Menyuan earthquakes. Geophys. Res. Lett..

[31] Feng W., He X., Zhang Y., Fang L., Sergey S., Zhang P. (2022). Seismic faults of the 2022 Mw 6.6 Menyuan, Qinghai earthquake and their implication for the regional seismogenic structures. Chin. Sci. Bull..

[32] Li Y., Jiang W., Li Y., Shen W., He Z., Li B., Li Q., Jiao Q., Tian Y. (2022). Coseismic Rupture Model and Tectonic Implications of the January 7 2022, Menyuan Mw 6.6 Earthquake Constraints from InSAR Observations and Field Investigation. Remote Sens..

[33] Bekaert D., Hooper A., Wright T.J. (2015). A spatially variable power law tropospheric correction technique for InSAR data. J. Geophys. Res. Solid Earth.

[34] Bai X., Zhang B., Xu H., Zhan X., Yan Y. (2023). Atmospheric Error Correction of InSAR Co-Seismic Deformation Based on DEM and GACOS. J. Geod. Geodyn..

[35] Wessel P., Smith W.H.F. (1998). New, improved version of generic mapping tools released: EOS. Trans. Am. Geophys. Union.

[36] Sandwell D., Mellors R., Tong X., Wei M., Wessel P. (2011). Open Radar Interferometry Software for Mapping Surface Deformation: EOS. Trans. Am. Geophys. Union.

[37] Chen C.W., Zebker H.A. (2000). Network Approaches to Two-Dimensional Phase Unwrapping: Intractability and Two New Algorithms. J. Opt. Soc. Am. A Opt. Image Sci..

[38] Rosen P., Gurrola E.M., Sacco G., Zerbker H.A. (2011). InSAR Scientific Computing Environment—The Home Stretch.

[39] Xu X., Tong X., Sandwell D.T., Milliner C.W., Dolan J.F., Hollingsworth J., Leprince S., Ayoub F. (2016). Refining the shallow slip deficit. Geophys. J. Int..

[40] Mario C. (1998). A novel phase unwrapping method based on network programming. IEEE Trans. Geosci. Remote Sens..

[41] Zhou Z., Liu L., Jiang L., Feng W., Samsonov S.V. (2019). Using Long-Term SAR Backscatter Data to Monitor Post-Fire Vegetation Recovery in Tundra Environment. Remote Sens..

[42] Li Z., Feng W., Xu Z., Cross P., Zhang J. (2008). The 1998 Mw5.7 Zhangbei-Shangyi (China) earthquake revisited: A buried thrust fault revealed with interferomertic synthetic aperture radar. Geochem. Geophys. Geosyst..

[43] Li Z., Elliott J.R., Feng W., Jackson J.A., Parsons B.E., Walters R.J. (2011). The 2010 Mw 6.8 Yushu (Qinghai, China) Earthquake: Constraints provided by InSAR and Body Wave Seismology. J. Geophy. Res. Solid Earth.

[44] Feng W.P., Xu L.S., Li Z.H. (2010). Fault parameters of the October 2008 Damxung Mw6.3 eartqhuake from InSAR inversion and its implication. Chin. J. Geophys..

[45] Feng W.P., Li Z.H. (2010). A Novel Hydrid PSO/Simplex algorithm for determining earthquake source parameters using InSAR data. Prog. Geophys..

[46] Jónsson S., Zebker H., Segall P., Amelung F. (2002). Fault Slip Distribution of the 1999 Mw7.1 Hector Mine, California Earthquake, Estimated from Satellite Radar and GPS Measurements. Bull. Seismol. Soc. Am..

[47] Okada Y. (1985). Surface deformation due to shear and tensile faults in a half-space. Bull. Seismol. Soc. Am..

[48] Fukahata Y., Wright T.J. (2008). A non-linear geodetic data inversion using ABIC for slip distribution on a fault with an unknown dip angle. Geophys. J. Int..

[49] Toda S., Stein R.S., Richards-Dinger K., Bozkurt S.B. (2005). Forecasting the evolution of seismicity in southern California: Animations built on earthquake stress transfer. J. Geophys. Res..

[50] Lin J., Stein R.S. (2004). Stress triggering in thrust and subduction earthquakes, and stress interaction between the southern San Andreas and nearby thrust and strike-slip faults. J. Geophys. Res..

[51] Institute of Geology and Lanzhou Institute of Seismology (IGLIS) (1993). The Qilianshan–Hexi Corridor Active Fault System.

[52] Taylor M., Yin A. (2009). Active structures of the Himalayan-Tibetan orogen and their relationships to earthquake distribution, contemporary strain field, and Cenozoic volcanism. Geosphere.

[53] Xu X., Han Z., Yang X. (2016). Seismotectonic Map in China and Its Adjacent Regions.

[54] Klinger Y., Xu X., Tapponnier P., Van der Woerd J.R., Lasserre C., King G. (2005). High-resolution satellite imagery mapping of the surface rupture and slip distribution of the Mw~7.8, 14 November 2001 Kokoxili earthquake, Kunlun Fault, Northern Tibet, China. Bull. Seismol. Soc. Am..

[55] Liu Z., Yu C., Li Z., Zhang X., Zhang M., Feng W., Han B., Peng J. (2023). Co- and post-seismic mechanisms of the 2020 Mw 6.3 Yutian earthquake and local stress evolution. Earth Space Sci..

