# Supplementary material for: Source Models of the 2016 and 2022 Menyuan Earthquakes and Their Tectonic Implications Revealed by InSAR
Source: Sensors (Basel). 2024 Jun 4;24(11):3622. doi: 10.3390/s24113622 (PMC11175176; doi:10.3390/s24113622)
Supplement: Supplementary file 1 [file sensors-24-03622-s001.zip › sensors-2846873-supplementary.pdf]

Supporting Information for

# Source Models of the 2016 and 2022 Menyuan Earthquakes and their Tectonic Implications Revealed by InSAR

**Xixuan Bai[1], Bingqiang Zhang[1], Aizhi Guo[2], Yi Yan[4], Hao Xu[2,3], Xiaoya Bian[1], Shuwen Zhan[1], Jiangcheng Chen[1]**

1. School of Civil Engineering and Architecture，Wuhan Institute of Technology，693 Xiongchu Avenue, Wuhan 430074，China;
2. State Key Laboratory of Geodesy and Earth’s Dynamic, Innovation Academy for Precision Measurement Science and Technology, Chinese Academy of Sciences, 340 Xudong Street, Wuhan 430077, China;
3. College of Earth and Planetary Sciences, University of Chinese Academy of Sciences, 1 Yanqihu East Road, Beijing 100049, China;
4. College of Resources and Environment, South-Central Minzu University 430074, China

*. Correspondence: wit_bianxy@hust.edu.cn;

# Introduction

In the supporting information, we present the result of atmospheric error correction, the coherence of radar signals requires that the coherence of the red inside of the mask is much lower than that of the surrounding area, the data processed using snaphu algorithm, coseismic deformations after down-sampling, the optimal dip angle of F2 in a multi-fault model for the 2022 earthquake. The Tab. S1 shows the statistical parameters of 2016 Menyuan earthquake coseismic deformations error before and after correction.

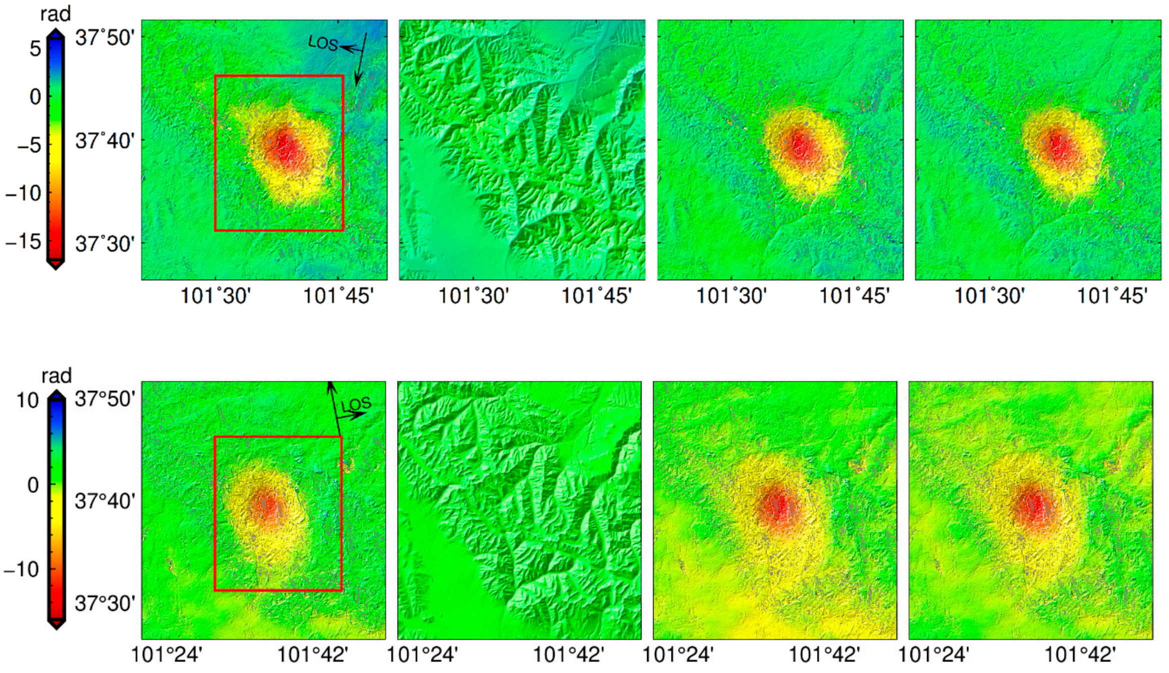

**Figure S1.** The function model and result of atmospheric error correction, (a) is the fitting function of the ascending (T128), (b) is the fitting function of the descending (T33), and the first from left to right in the result is the initial observation, the atmospheric error model, the atmospheric error correction result and the observation after the fitting trend. The regions in the red rectangle are subtracted when fitting the function.

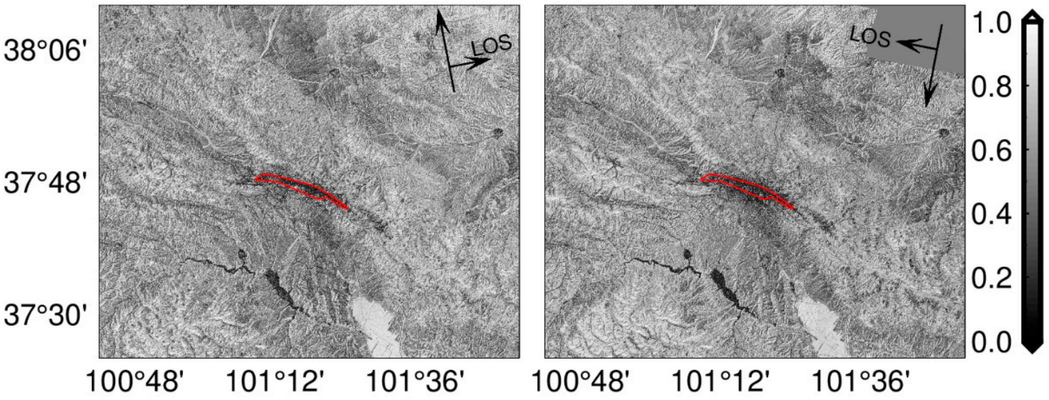


**Figure S2.** The coherence of radar signals requires that the coherence of the red inside of the mask is much lower than that of the surrounding area.

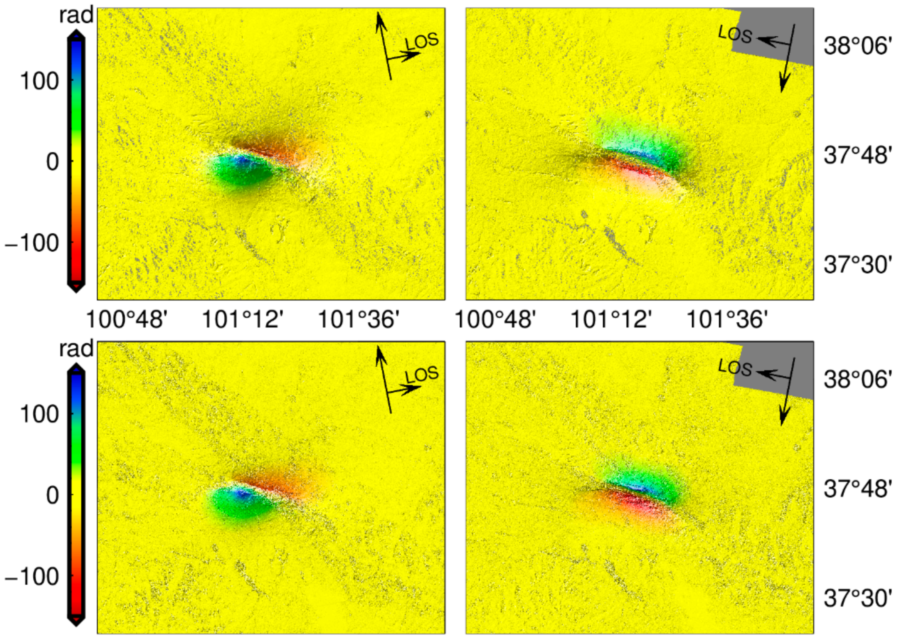


**Figure S3.** Results of processed using snaphu algorithm ;(a) Observations of the software GMTSAR; (b) Observations of the software ISCE.

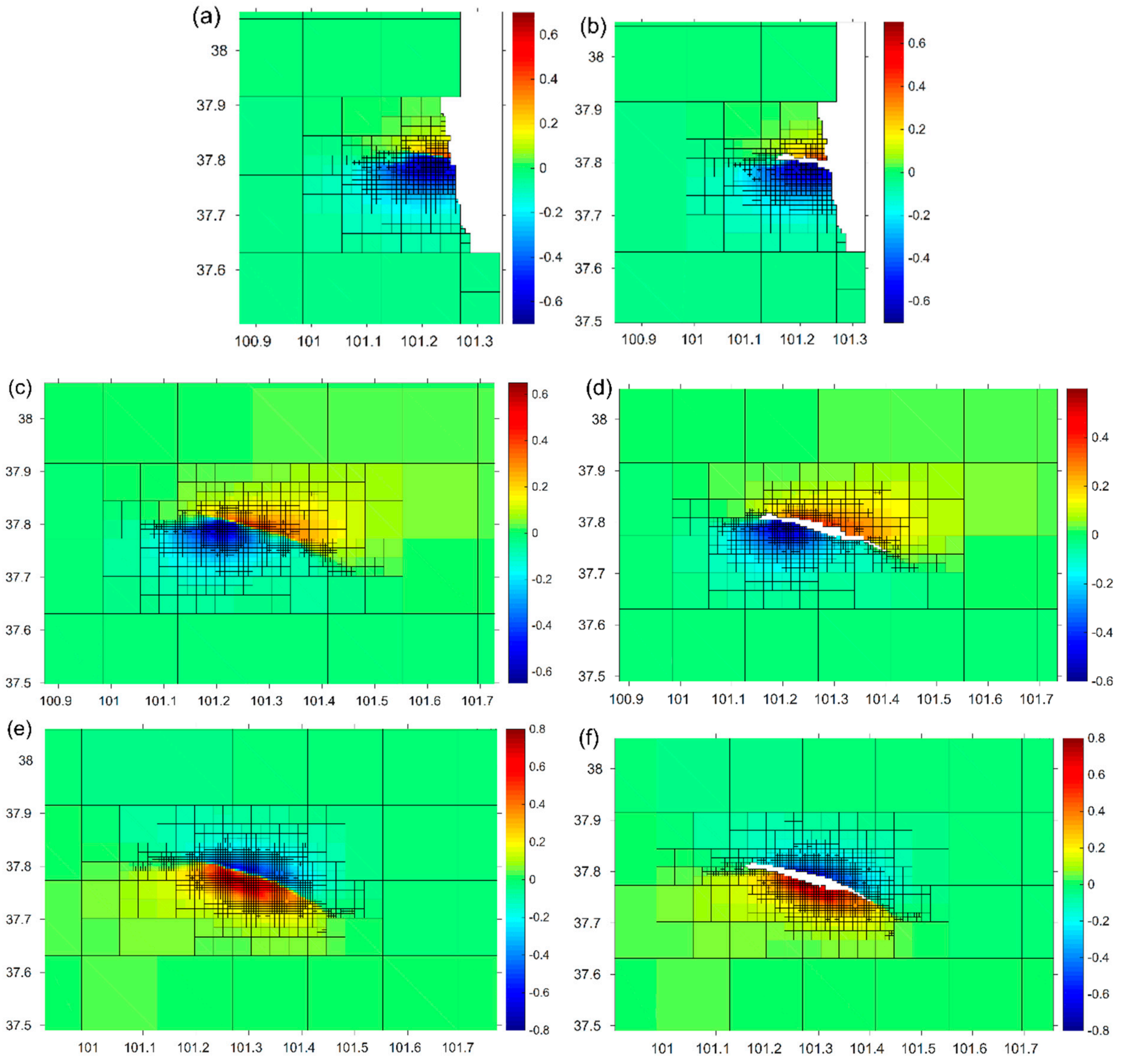


**Figure S4.** Coseismic deformations after down-sampling; (a), (c), (e) are the observations before the low coherence region is masked, and (b), (d), (f) are the observations after the mask.

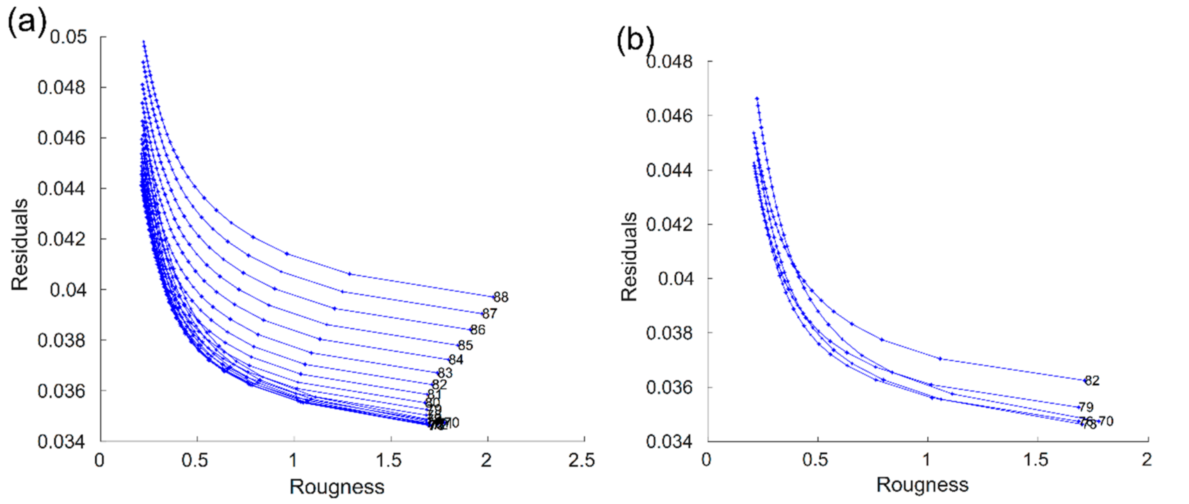

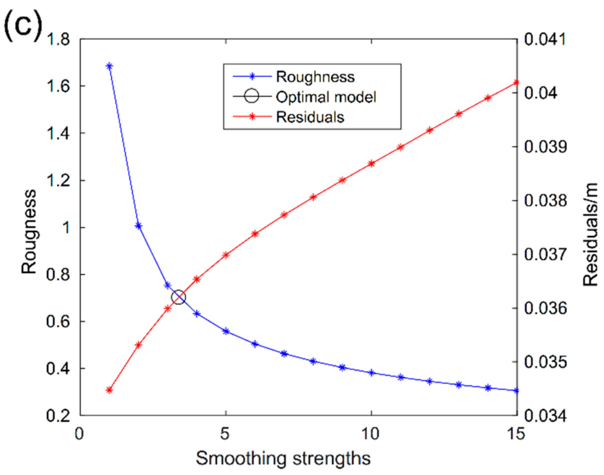


**Figure S5.** The optimal dip angle of F2 in a multi-fault model for the 2022 earthquake; (a) is the residual and roughness corresponding to all the tested models; (b) The corresponding parameter when the dip angle is less than 82o, in order to avoid data density, the interval is set as 3o; (c) Optimal model corresponding to inclination 81o.

**Table S1** Statistical parameters of 2016 Menyuan earthquake coseismic deformations error before and after correction

| | Before correction | | | After correction | | | |
|---|---|---|---|---|---|---|---|
| Track | Coe | RMS/cm | Sd/cm | RMS1/cm | RMS2/cm | Sd1/cm | Sd2/cm |
| T128 | 0.25 | 2.025 | 1.671 | 1.861 | 1.727 | 1.610 | 1.480 |
| T33 | 0.76 | 2.373 | 2.157 | 2.066 | 2.011 | 1.779 | 1.717 |
